# Domestication affects exploratory behaviour of pikeperch (*Sander lucioperca* L.) during the transition to pelleted food

**DOI:** 10.1371/journal.pone.0196118

**Published:** 2018-05-09

**Authors:** Tamás Molnár, Adrienn Csuvár, Ildikó Benedek, Marcell Molnár, Péter Kabai

**Affiliations:** Institute of Environmental Science and Nature Protection, Kaposvár University, Kaposvár, Hungary; Institut National de la Recherche Agronomique, FRANCE

## Abstract

Genetic selection for body size during domestication of animal species can inadvertently affect a number of physiological and behavioural traits. The pace-of-life syndrome (POLS) hypothesis predicts that domestication in an artificial environment lacking predators and providing abundant resources prefers proactive individuals with strong feeding motivation, high levels of aggression and risk taking, with low hypothalamus–pituitary–adrenal (HPA) axis responsiveness. In the present experiment we weaned fingerling pike-perch from live feed and habituated them to formulated feed. We recorded the number of weeks needed for the fish to accept pellets, their body length at the age of 100 days, their boldness in a novel object test and their HPI axis responsiveness. Individuals accepting the artificial feed within the first week grew larger than fish habituating later; therefore early weaners would be kept and bred in routine aquaculture procedures. Contrary to predictions of POLS hypothesis, fish weaning earlier and thus growing faster were less bold and had higher HPI axis responsiveness than fish accepting the pellets later or never. As live feed is preferred to artificial pellets, less competitive individuals may switch to pellets earlier. Inadvertent selection for stress sensitive fish may have an effect on production in aquaculture as well as on natural population after intensive restocking.

## Introduction

Foraging in a habitat rich in food may be at trade-off with high predatory pressure and intraspecific competition. An alternative adaptive response to such challenges can be to forage at patches with less food, competitors and predators, as there might be more than a single optimal solution, alternative strategy can be present in a population [1].

Several theories have addressed the issue of divergent responses to challenges. According to Koolhass et al. [2] behavioural and physiological characteristics consistently associated under stressful conditions represent coping styles, defined as joint behavioural and physiological stress responses that are stable over time and which are characteristic to a certain group of individuals [2]. There may be several coping styles in a population, the two extremes being proactive and reactive. At the physiological level proactive strategy is associated with low hypothalamus–pituitary–adrenal (HPA) axis responsiveness whereas HPA response is very high in reactive individuals. As behavioural traits can be consistently associated in non-stressful situations as well, more general terms such as personality or behavioural syndromes were introduced and are now used basically as synonyms of coping style or the classical terms such as temperament or emotionality. The two extreme coping styles are placed on the opposite sides of five personality axes recognised in animals.

Metabolic rate was considered as an explanatory variable for differences in coping styles first in comparison of species and later in intraspecific comparison in the pace-of-life-syndrome (POLS) hypothesis. An extension of the pace-of-life syndrome (POLS) hypothesis (reviewed by Réále et al. [3] proposes that physiological traits and life history should be closely coupled to behavioural traits. Individuals with higher metabolism need more energy intake, grow faster and therefore follow a fast pace of life compared to slow pace animals. In consequence fast pace individuals are supposed to be bold explorers and aggressive with low HPA reactivity. Concerning fish, a number of studies consistently showed that individuals with a fast pace of life have higher feeding motivation, oxygen consumption, risk taking, aggressiveness and lower HPI reactivity compared to slow pace individuals (reviewed by Castanheira et al.) [4].

The process of domestication selects animals with high growth rate therefore it seems to favour fast pace individuals. Genetic selection pressure for adaptation to the man-made environment is high in the culture conditions which are very different from the natural environment [5]. Intensive indoor aquaculture is one example of an extremely artificial environment. Fish are kept in aerated tanks without vegetation or hiding places to ensure free flow of the water, accessible food is provided and in a predictable pattern, predators are absent, and rearing density is significantly higher than in nature. Such alterations from the natural environment are expected to bring behavioural and physiological adaptations with them. Albeit, the repeatability and heritability of glucocorticoid stress respond has been already confirmed [6] intra- individual variability of stress induced cortisol levels could be condition dependent [7, 8]. Absence of appropriate shelter induces increase in standard metabolic rate coupled with changes in colouration indicating greater stress [9]. Social status could also modify the HPI axis function. In subordinate fish resting cortisol level was elevated but plasma cortisol response failed to an acute stressor in rainbow trout (*Onchorhynchus mykiss*) [8, 10]. This attenuated stress response driven by the aggressive competition shows recovery during 48–96 hours after separation of the subordinate individual. Plasma cortisol level returns to baseline value and the fish exhibit “normal” HPI response to an acute stressor. Although, recovered individual will achieve only subordinate status in the hierarchy in the future [11].

Genetic selection for body size in the absence of predators might diminish anti-predatory behaviour [12, 13] and thus increase boldness [14] and lower HPI reactivity [15]. It is less clear how exploration and aggression might be affected by domestication, because exploratory behaviour is a trade-off of at least two opposing tendencies: learning about the environment to exploit it and staying safe from predators. In the artificial environment there is no predatory pressure, however, the abundance of resources does not necessitate exploration. Similarly, it is not clear how agonistic tendencies might change through domestication. Fast pace individuals are supposed to be highly competitive and aggressive, however, aggression can be a major source of mortality, therefore breeders aim to reduce the level of overt aggression by optimal fish density [16, 17], frequent feeding, or by reducing the opportunity to monopolise food by feeding from numerous feeders or making feeding time unpredictable (reviewed by Magnhagen) [18]. When agonistic behaviour does not help to obtain resources, the cost of aggression is higher than its benefit; therefore aggression might be inadvertently selected against. The dependence of cost and benefit of agonistic behaviour on technology and species might explain the contradiction in reports of increasing or diminishing aggression through domestication (discussed by Brelin et al.) [19].

In the present study we asked how traits connected to pace of life, such as HPI reactivity and boldness would be co-selected when selecting for body size of a predatory and cannibalistic fish species, the pikeperch (*Sander lucioperca* L.), in an intensive rearing system. Here fish are trained to accept pelleted food instead of the preferred live prey. Such training can be carried out at fingerling stage which is when in nature the transition from zooplankton to fish prey consumption would happen. Transition to pelleted food at the fingerling stage may cause 14 to 59% mortality [20] indicating strong selection pressure induced by weaning during the first few generations of domestication.

## Material and methods

### Ethical approval

All procedures were in accordance with the ethical standards of the institution and followed the European Directive 2010/63 UE. The protocol was approved by the Committee on the Ethics of Animal Experiments of the Kaposvár University (permit number: 3/2016-MÁB). All fish handling procedures (except just before behavioural tests) were conducted under anesthesia to minimize animal stress and suffering.

### Culture facility

The rearing unit contained thirty 65 L (60 cm _30 cm _ 30 cm, L _W _ H), aerated aquaria as parts of a recirculation system. This system had a total volume of 2500 L (attached to a simple bio-filter unit. The daily water replacement rate was about 10% of the total volume. The water flow rate was adjusted to 1.5 L min^-1^ and the temperature was kept at 21 ± 0.5°C.

### Experimental fish

Juvenile pikeperch, (*Sander lucioperca* Linneaus 1758) (mean standard length 38.4±0.6 mm, N = 2000) were purchased from BO-FA Fish Farm (Attala, Hungary). The used population has its origin in Lake Balaton and it has been bred under pond culture conditions using hormone induced propagations in the last 10 years. Targeted selection was not performed, but spontaneous genetic selection was demonstrated in two microsatellite markers in the broodstock. The weaning selection happened in the first generation. The brood fish were hormonally treated and stripped, the fertilised eggs were incubated; fry rearing was carried out in small ponds. At the age of 35 days juveniles were transported to the Breeding Facility of the Department of Aquaculture and Fishery at Kaposvár University.

### Feeding trial

Altogether 2000 individuals were introduced to eight 300L aquaria (fish density was 0.8 individuals/L). In the first and second day fish were fed minced *Tubifex* ad libitum twice a day (8 AM and 6 PM). Transition from live food to pelleted feed was encouraged by mixing minced *Tubifex* with pellets from the third day [21]. Initially, *Tubifex* and the pellets were mixed at equal amounts, then the ratio of *Tubifex* was gradually decreased while the size of the pellets was increased from 1.5 mm to 3 mm. Pellets consisted of 54% crude protein, 18.1% crude fat and 27.9% extractable non-nitrogenous compound.

Portions of the feed were dropped into the tank one by one and feeding tactics of the fish were observed.

During the 3 weeks the same feeding regime was performed for all fish. However, to identify and separate individuals accepting pelleted feed, tests were done on 3 days. On day 7, 14 and 21 of the feeding trial only pellet was offered until individuals accepting the pelleted food were satiated. Pellet consumption was recognised by the yellow figuration of the abdomen. Fish accepting the pellet were removed and grouped in separate tanks as switching to pellet “Early weaners” (day 7), “Normal weaners” (day 14), or “Late weaners” (day 21). Fish not accepting the pellet in 3 weeks were grouped as “Non-weaners”. Non-weaners were provided with *Tubifex* only. Tanks were checked twice daily for cannibalistic individuals which were separated at once and used as an additional treatment group (Cannibals). Assessing cannibalistic individuals happened on the recognition of the “double sized stomach” or the tail of the ingested fish hang out of the cannibalistic individual.

### Novel-object test

The novel object test was performed according to the method described by Jones and Godin (2010) [22]. At the age of 90–110 days standard body length of 24 Early weaning, 24 Normal weaning, 24 Late weaning, 14 Cannibals and 70 Non-weaning fish were measured and the fish were transferred into test tanks (50 cm _30 cm _ 30 cm, L _W _ H) individually. Following 24 hours of habituation a video camcorder (Sony HDR-XR) was started and a small yellow Lego block (LEGO 6176 DUPLO Basic Brick) attached to a metal sinker was placed in the centre of the tank as a novel object and the behaviour of the fish was recorded for 30 min. The number of approaches to the novel object, closest distance from the object and latency of the closest approach were estimated.

### Cortisol assessment

From the Early, Normal and Late weaning groups 17-17-17 individuals were subjected to confinement stress by keeping them in a net (15x20 cm) for 5 min. After the confinement the fish were anesthetized with clove oil (10 drops in 10 L water). Blood samples were taken 12–15 minutes after the start of the confinement, from the caudal vasculature to heparin-coated tubes, centrifuged (10000G/2 min) and the plasma was stored frozen (-80°C) until analysed. Blood serum cortisol concentrations were measured by radioimmunoassay (RIA) method with Kortizol [^125^I] RIA kit (Isotope Institute Ltd., Budapest, Hungary) and gamma counter [23]. Intra and interassay variability were (CV%) 5.47% and 6.55%, respectively.

### Statistical analyses

Shapiro-Wilk test showed the data was not normally distributed therefore nonparametric statistics were used. A k-means cluster analysis (with k = 3, iteration number = 10) was performed on the novel-object test variables to categorise the individuals as explorative, average or retractile. The distribution of the clusters in the five feeding groups (Cannibal, Early, Normal, Late, and Non-weaning) was analysed by Mann-Whitney U test. Bonferroni correction was applied in all multiple comparisons of the five groups by setting critical P value at 0.01 (0.05 divided by 5). Individual cortisol levels in the three pellet feeding groups (Early, Normal, Late) were analysed by One-way ANOVA using post-hoc Tukey test. Statistical analyses were conducted using SPSS Statistics 11.5 (2002).

## Results

### Feeding and growth

**Fig 1:**
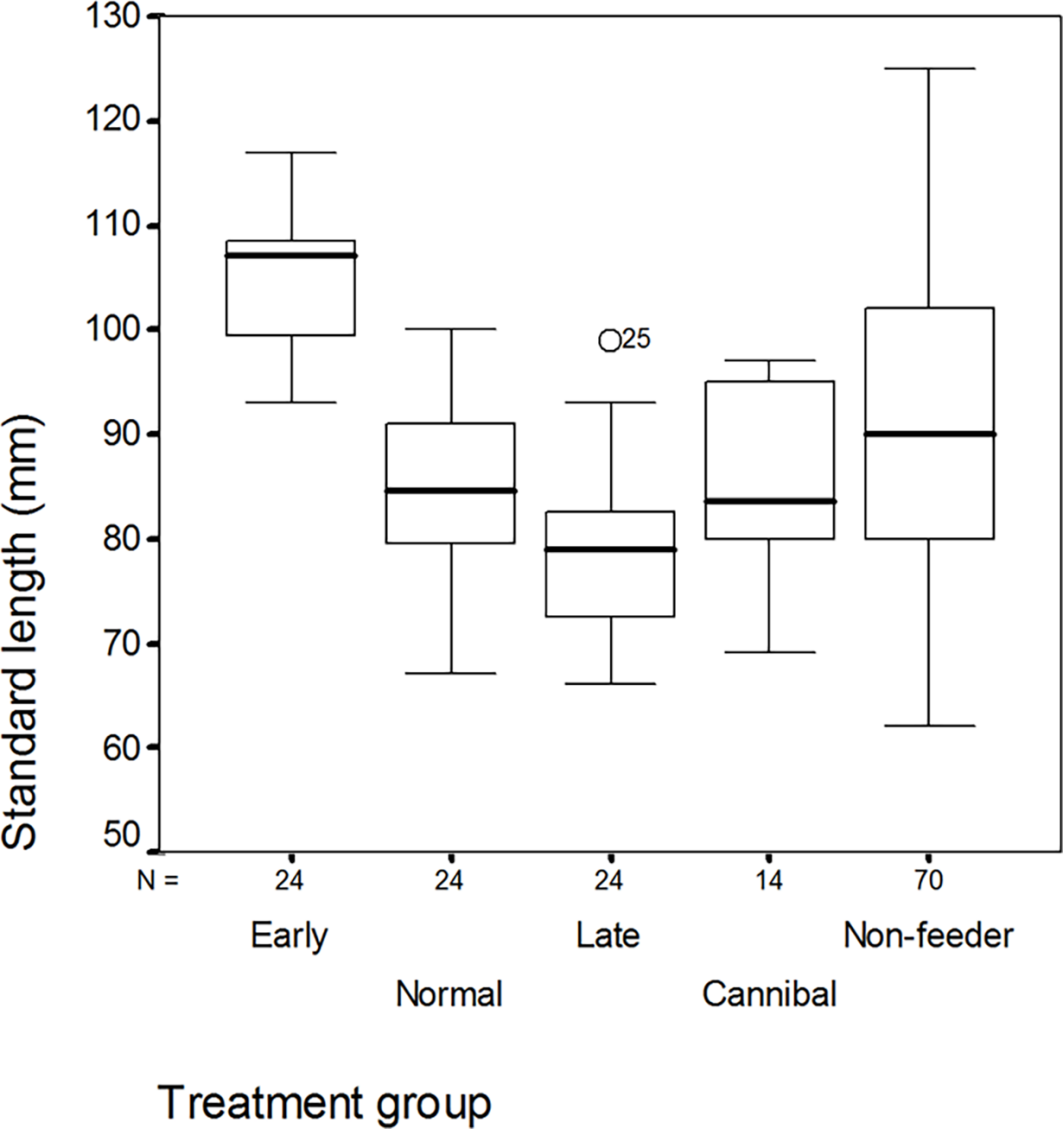
Variation in the standard length in the groups formed by the time of starting on pellet feeding. Different letters above the columns indicate significant differences (Mann-Whitney U test, P<0.01).

The nutritional value of the pelleted food is higher than that of *Tubifex*, therefore fish accepting pellets earlier grew faster. Early weaners (104.8±6.5mm) had significantly higher body length compared to the Late-weaning group (78.9±8.3 mm) which differed from Non-weaners (91.1±13.6 mm) as well. Normal (85.0±8.0 mm) and Cannibal (85.2±9.1 mm) groups showed intermediate size (Fig 1).

The three feeding tactics observed were in congruence with previous findings by Molnár et al. [24]. When feeding was started some individuals gathered and stayed at the front of the tank, others stayed in the darker background, darted for the feed and withdrew immediately regardless of success, and thirdly, some fingerlings did not attack the feed close to competitors but picked up abandoned feed even from the floor.

### Novel object test

There were some clear tendencies in the novel-object test variables across the feeding groups. Exploration in terms of closest distance from the novel object, short latency of closest approach and high number of approaches seem to be at a lower level in the Early-weaning group and high in the groups which accepted pellets later or never. Cannibalistic fish behaved similarly to the Early-weaning group. Because of the high variation within the groups and the non-normal distribution of the data the pattern of significance is complex (Table 1, S1 Table).

**Table 1:** Differences among the feeding groups in measured variables of exploratory behaviour.

Treatment group	Early	Normal	Late	Cannibal	Non-weaning
Latency (min)	18.4±11.3^ab^	16.5±9.7^ab^	10.6±7.3^a^	21.7±10.2^b^	13.2±8.8^a^
Closest distance (cm)	13.5±9.4	10.9±5.7	8.9±6.6	12.8±8.4	8.9±5.8
Number of approaches	2.3±2.8^a^	1.7±1.9^a^	4.8±3.9^b^	2.5±2.7^ab^	4.7±3.5^b^

Values labelled by different letters are significantly different at P < 0.01

**Fig 2:**
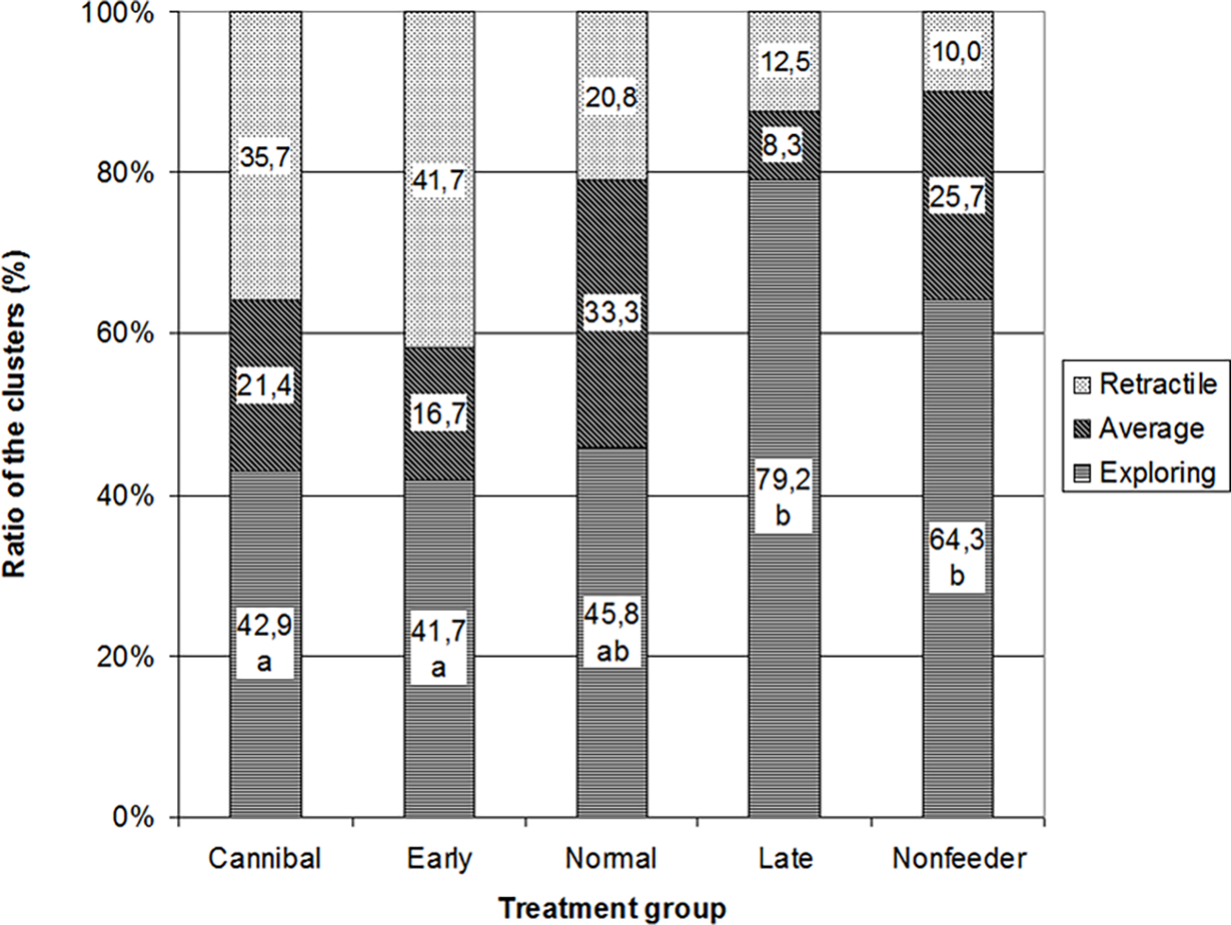
Proportion of juvenile pikeperch assigned to 3 clusters (explorative, average or retractile) in the feeding groups. Different letters above the columns indicate significant differences (Mann-Whitney U test, P<0.01).

We performed k-means cluster analysis (k = 3) within groups on all three variables reducing the complexity of the data to three clusters of higher, average or lower exploration variables (explorative, average or retractive, respectively, S1 Table). Feeding type had significant connection with the ratio of exploration levels among the groups (Kruskal-Wallis,χ2(4) = 13,326 P<0.05). Post-hoc Mann-Whitney U test revealed that Cannibal and Early weaning groups contained significantly fewer explorative individuals than Late and Non-weaning groups (for levels of significance see Fig 2).

Analysis of variance on the cortisol levels yielded significant variation among pellet feeders (One-way ANOVA F(2,48) = 4.628, P = 0.015). Post-hoc Tukey test revealed significant difference between Early and Late weaners (255.5± 44.2 ng/ml and 175.8± 78.6 ng/ml, respectively, P < 0.01) whereas Normal weaners had intermediate cortisol levels (217.4 ±96.8 ng/ml) which are statistically not different from the two other groups.

Among the three investigated factors (feeding type, explorative behaviour and cortisol level) only the feeding type proved to have significant effect on the standard length (Table 2).

**Table 2:** Relation of feeding type, explorative behaviour and cortisol level as fixed factors with standard length as dependent variable analysed by general linear model.

Source	Sum of Squares	df	Mean Square	F	Sig.
Corrected Model	7891.543	9	876.838	18.485	0.001
Intercept	23798.750	1	23798.750	501.719	0.001
Cortisol level	46.113	1	46.113	0.972	0.330
Treatment group	5066.044	2	2533.022	53.401	0.001
Exploration Cluster	13.177	2	6.588	0.139	0.871
Exploration Cluster * Treatment group	224.008	4	56.002	1.181	0.334
Error	1944.810	41	47.434		

Individual cortisol level was used as covariant. (R^2^ = 0.802, Adjusted R^2^ = 0.759)

## Discussion

Cross-context analysis of the behaviour of juvenile pikeperch [25] assessed the presence of behavioural syndromes. This species responded in the same way to exploration and dyadic test but their responses were opposite in the restraint test. The objective of this study was to ascertain whether HPI reactivity and boldness would be co-selected either with learning ability or with body size? As for unexpected results of the present study we found, that pikeperch individuals accepting the pelleted food earlier were less bold and had higher level of stress-induced cortisol than fish which switched to the artificial feed later or never accepted the pelleted feed. The explanation for our results could be found in the process of weaning. Fingerlings follow three distinct tactics when meeting the mixture of live and artificial feed. Some fish stay motionless close to the surface and attack the feed in their vicinity. Other individuals stay in the darker background, dart for the feed when dropped in the water and withdraw back to the background. Fish following such tactics aim at the live prey and might either swallow or eject pellets which have accidentally got into their mouths. The third tactic is opportunistic feeding; such individuals eat whatever is left by the others, in most cases the non-preferred pellets. Opportunistic feeders would pick up feed even from the bottom of the tank [21, 24]. As the nutritional value of the pellets is higher than that of *Tubifex*, fish accepting the artificial feed earlier grow larger, while individuals rejecting the pellets would not survive in intensive aquaculture practice.

In case of percid fish the ontogenetic changes in foraging (switching to piscivory) occur when the net energy intake is higher on the new diet and the potential prey (fry or larvae) is available in an adequate density [18]. Naïve pike-perch would not readily eat pelleted feed when offered for the first time. To overcome such neophobia pellets are mixed with familiar live food when introduced. The fish usually targets the live prey but might bite accidentally on the pellets raising the probability of familiarising with the novel feed [21, 26] and accepting it when the ratio of live food is significantly reduced. Increased energetic demand with limited resources (as it occurs during the weaning to pelleted food) may require an increased tendency to try new routes to obtain enough food in fish [14]. Hatchery reared brown trout (*Salmo trutta*) learn to reduce its search time for cryptic prey compared to wild which performs higher predator vigilance [27]. Less explorative (shy) individuals has higher behavioural flexibility in agreement with the theory on stress coping styles, that reactive individuals adjust their behaviour more rapidly according to the environmental changes [28].

Although we did not estimate dominance hierarchy during transition, the pattern of competition clearly suggests that pellets were accepted earlier by the less competitive, opportunistic feeders. The early transition group tended to be shyer and had higher stress induced cortisol level than fish that accepted the pellets later. Since the landmark study of Huntingford [29] on three-spined stickleback the possible connection between dominance and boldness has been documented in other fish species, for example in brown trout [14], rainbow trout [30, 31], and grayling [32]. The connection between boldness and low cortisol response suggested by our results are in line with the studies on two strains of rainbow trout genetically selected for high or low cortisol responsiveness to a standardized stressor (see review [33]). Fish with low cortisol responsiveness were socially dominant over highly responding fish [34, 35] and were bolder in an unfamiliar environment [31] and resumed feeding in a novel environment sooner than did fish from the highly responding strain [36].

Another unexpected result is that cannibalistic fish were as shy as Early weaners in the novel object test. The present study did not focus on cannibalism; fish were labelled as cannibals upon a single event of intraspecific predation. In accordance with aquaculture practice, cannibalistic fish were removed as soon as recognised but instead of discarding them they were treated as an additional experimental group. Intraspecific predation is generally linked to aggression (reviewed by Naumovicz et al.) [37] in an environment where resources can be monopolised therefore dominant individuals can eat more and grow faster [18] than subordinates. However, similar to the study on vundu catfish [38] in the present experiment no agonistic behaviour was observed before predation. Cannibalistic events almost exclusively happened during feeding time when attacks were preceded by the sudden darting of fish toward the food. We speculate that fish unsuccessful in competition for live feed either accepted pellets or opted for intraspecific predation which could explain similar shyness of Early weaners and Cannibals in the novel object test. Such hypothesis will be tested in future studies.

Selective breeding causes targeted changes in the genetic composition of farmed populations (e.g. faster growth, increased disease resistance) as well as inadvertent changes as natural selective pressures are reduced in the artificial environment (e.g. lower cortisol and glucose levels, increased aggression and diminished anti-predatory behaviour) [39]. Several studies suggest that boldness, anti-predatory behaviour as well as stress responses are highly heritable traits e.g. [7, 40, 41, 42], predicting strong response to genetic selection in the first few generations [5]. The pace of life syndrome (POLS) hypothesis predicts that fast pace individuals would be favoured when selecting for body size during domestication. However, there are exceptions where bold individuals showed no correlation with growth during hatchery rearing [14] or shy individuals selected under hatchery conditions performed higher growth rate in the wild [28]. Our results indicate an opposite effect of selection in the case of artificially fed pikeperch supposing the consistency of the behaviour over time. Early transition to the more nutritious pelleted feed resulted in bigger body size in shy individuals with high HPI reactivity. Fluctuations in predation risk and food ability are substantial factors in forming the direction and intensity of correlation between growth and personality [28]. Selection for rapid growth is often associated with domestic conditions including relaxed predation risk. However, weaning pikeperch to pelleted food is attended by high level of cannibalism either in larvae or fingerling age [43, 21], which means that predation risk occurs during this period. Concerning European seabass, *Dicentrarchus labrax* fish reared under unpredictable food supply showed bolder behaviour and realized lower growth rate compared to individuals reared on predictable food supply [44]. Early weaners were presumably opportunistic feeders resulting from more predictable food supply during the transition. Independently, the more flexible behaviour could result that feeding more nutritious pellet 7–14 days earlier resulted higher body weight without having higher growth capacity. Feeding trials with the different groups are needed to ascertain the real background.

## Conclusions

Genetic selection of artificially bred pikeperch would favour less bold and more stress sensitive early pellet eaters. Further studies (including growth studies over longer period of time) are needed to confirm our findings which may have yet unexpected consequences on the breeding and conservation of perch-pike. Production of fish with high stress sensitivity would be disadvantageous in aquaculture production as well as in nature where the large number of artificially selected fish might interbreed with the native population.

## Supporting information

S1 Table The raw data of the experiment. (XLS)
